# Sustainable entrepreneurship between ecological distinction and local context

**DOI:** 10.3389/fsoc.2026.1816778

**Published:** 2026-07-08

**Authors:** Laura Azzolina, Sarah Mazzenzana

**Affiliations:** Dipartimento di Scienze Politiche e delle Relazioni Internazionali (DEMS), University of Palermo, Palermo, Italy

**Keywords:** Palermo, peripheral regions, place-based enterprise, pro-environmental attitudes, sustainability transitions, sustainable development, sustainable entrepreneurship

## Abstract

Long regarded as the primary driver of the destructive effects associated with globalized and neo-liberal capitalism, in recent decades firms have re-emerged in scholarly debates not only as a source of problems but also as an agent for promoting more inclusive and environmentally sustainable development models. The growing diffusion of new business models calls for explanations concerning their origins, motivations and intentions, as well as the role of the contexts, factors that ultimately shape their capacity to support concrete transition processes. This paper integrates categories drawn from the literature on sustainable entrepreneurship and from ecological distinction applied to pro-environmental attitudes into a unified analytical framework, with the aim of improving our understanding of emerging forms of entrepreneurship while accounting for territorial specificities. The empirical basis of the research was developed within the case study of Palermo, one of the largest urban areas in southern Italy, which is a peripheral region of Europe. The research employs a qualitative methodology through the use of discursive interviews conducted with 24 entrepreneurs in the service sector, mainly food, circular economy, and sustainable tourism. The paper proposes a typology of firms that makes it possible to highlight the features of a hybrid form of entrepreneurship, interpreted in light of its social foundation (the urban critical middle class) and the characteristics of the territory in the case study (a peripheral area).

## 1 Introduction: exploring the social and territorial conditions of sustainable entrepreneurship

Directing growth toward sustainable development goals, with reference to both the social and environmental dimensions, is among the most urgent challenges that cities are currently facing, as established by the United Nations 2030 Agenda for Sustainable Development (SDG 11) and reiterated by the subsequent New Urban Agenda. Within this framework, in the extensive literature on sustainability and ongoing transitions, the idea that businesses can represent part of the solution—rather than simply the cause—of environmental degradation and social inequalities (Muñoz and Cohen, 2017) has gained increasing importance. While in the early phase of globalization the pursuit of indiscriminate growth by the traditional competitive firm, driven by profit-maximizing entrepreneurs, was considered the main driver of “market fundamentalism” (Block and Somers, 2014) and its destructive effects on the environment and society, in recent decades new business models and entrepreneurial practices have emerged, characterized by economic actions more attentive to environmental issues and social relationships. These developments call for explanations regarding their origins and their potential for diffusion.

This contribution focuses on sustainable entrepreneurship as a relevant actor in advancing the transition process.

On this topic, organizational studies have provided a particularly significant contribution in defining the various unconventional configurations of firms and sustainable entrepreneurship. However, they have devoted relatively less attention to other aspects, such as their genesis and the role of the contexts in which they develop, thereby opening the way for further research directions. Here, two research questions are addressed. First, how do these different entrepreneurial models emerge? Second, in which territorial contexts can they spread? These are issues of great theoretical and practical-political relevance.

Theoretical approaches developed mainly within the sociology of consumption argue that orientations and behaviors consistent with sustainability, such as pro-environmental attitudes, function as emerging practices of distinction among different social groups (Flemmen, 2025). This suggests the usefulness of adopting a perspective capable of capturing the social—rather than individual—genesis of entrepreneurial orientations as well.

A first specific objective of this work is therefore to investigate which groups and which social mechanisms are relevant for the emergence of sustainable entrepreneurship.

On the other hand, it has also been shown that pathways toward sustainability are not homogeneous across space, but vary between territories also depending on their pre-existing level of development. This suggests that places differ in their capacity to pursue sustainability, highlighting the importance of adopting a place-sensitive analytical perspective in the study of transition processes (Rodríguez-Pose and Bartalucci, 2024). A second specific objective is therefore to explore to what extent sustainable entrepreneurial models can also emerge in contexts characterized by weak economic development.

Positioned at the theoretical intersection between studies on sustainable entrepreneurship, social distinction applied to environmental attitudes, and the literature on urban and regional transitions, and based on empirical research conducted in a peripheral, low-growth city—Palermo—this work proposes a deeper understanding of the social and territorial conditions underlying the development and diffusion of sustainable entrepreneurship.

The article is structured as follows: after presenting the literature review (Section 2) and the research method, dataset, and analytical criteria adopted (Section 3), the results are presented (Section 4). One subsection provides a mapping of firms within the urban territory, while another outlines the analytical framework used to organize and interpret the empirical material, leading to the presentation of different models of firms and entrepreneurship and the ways in which they promote sustainability. The concluding section (Section 5) discusses the findings in light of the existing literature and presents the final considerations.

## 2 Integrating three research streams on sustainable entrepreneurship: a literature review

The research stream on sustainable entrepreneurship, developed primarily within organizational studies, has made a significant contribution to the analysis of non-conventional models of entrepreneurship, recognizing firms as key actors in processes of sustainable transition. Rather than promoting a simplistic opposition between the stereotypical entrepreneur “driven by good intentions” and the traditional figure of the “homo economicus” (Halberstadt et al., 2024), this research stream has progressively placed non-conventional entrepreneurial configurations at the center of analysis. As has been observed, “sustainable entrepreneurship seems to be reaching a level of maturity as a subfield within the domain of entrepreneurship” (Muñoz and Cohen, 2017, p. 302). Since the forms and organizational profiles of these new models of entrepreneurship can vary considerably, this subfield has developed various analytical categories—sometimes with overlapping boundaries—to account for these differences.

From the historical model of the *Triple Bottom Line* (3BL), linked to the Brundtland Commission’s report “Our Common Future” (1987), which grounds sustainable development on the need to integrate environmental protection, economic development, and equity and from which an entrepreneurial model has emerged that integrates economic, social, and environmental goals into a holistic organizational system (Young and Tilley, 2006; Muñoz and Cohen, 2017) to the concept of *diverse economies* (Gibson-Graham, 2008); from *ecopreneurship*, to *social and institutional entrepreneurship* (Schaltegger and Wagner, 2011), to *purpose-driven urban entrepreneurship* (Cohen and Muñoz, 2015), understood as the articulation of collaborative public–private initiatives aimed at improving citizens’ urban well-being; and finally to *alternative economic practices*, that is, forms of economic coordination defined in opposition to capitalism (Sánchez-Hernández and Glückler, 2019). Among these, perhaps the category that has gained the greatest prominence is that of *hybrid organizations*, so defined because they focus not only on the economic dimension, but also on the social dimension—the creation of value for workers and/or communities external to the firm—and/or on the environmental dimension -the protection of ecosystems and the environment- (Martín-Rocañín et al., 2025).

Within the literature on sustainable entrepreneurship, alongside the now extensive evidence of the diverse configurations characterizing emerging entrepreneurial models, there is broad consensus that the development of these models is grounded in certain characteristics of the entrepreneurs themselves. In particular, the literature has emphasized two main traits: a strong value orientation, which enables entrepreneurs to identify social or environmental needs to be addressed through entrepreneurial action, and a sense of self-efficacy, that is, the belief that their actions can effectively counter environmental degradation and/or social fragmentation from which such needs arise (Muñoz and Cohen, 2017; Halberstadt et al., 2024).

However, some critical aspects of the debate emerge from the observation that sustainable entrepreneurs do not always start by identifying the needs of society and then providing an appropriate solution. Often the opposite occurs: entrepreneurs begin with the development of sustainable solutions and only subsequently seek out the needs that these solutions can satisfy (Halberstadt et al., 2024). This raises a key question: if entrepreneurial ideas do not originate from a reading of market demand, where do they come from? Can they be explained solely in terms of individual inclinations? Such considerations bring to the fore the unresolved theoretical issue of the genesis of sustainable entrepreneurship. In this respect, relying exclusively on individual values appears insufficient, thus calling for the integration of insights from other strands of research.

In this area, relevant insights emerge from the literature on environmental attitudes associated with different social groups. Starting from the observation that pro-environmental orientations in consumption choices are more widespread among higher social classes, some studies show that such preferences cannot be reduced to simple personal inclinations toward the environment, nor explained exclusively by economic status (Carfagna et al., 2014). Rather, they are interpreted as strategies through which groups endowed with high cultural capital construct their identity, assert their status, and distinguish themselves from other social groups. From this perspective, the concept of *eco-habitus* is employed, which, drawing on the Bourdieusian notion of habitus, is defined beyond individual orientations and in relation to social groups, taking on symbolic meanings in reference to other groups (Carfagna et al., 2014).

The concept of eco-habitus has given rise to a line of research -mainly within consumption theory- that places at its center the analysis of the distribution of pro-environmental attitudes across social classes and groups. Subsequent studies have shown, for example, that what distinguishes consumers from lower classes from those in higher classes is not necessarily a lack of environmental sensitivity, but rather a sense of ecological powerlessness (*eco-powerlessness*), understood as a feeling of resignation in contrast to the sense of self-efficacy typical of higher classes (Kennedy and Givens, 2019).

More recent contributions (Flemmen, 2025) confirm that environmental attitudes are stratified across social groups and that those with greater resources tend to exhibit stronger pro-environmental orientations. However, by distinguishing between different forms of capital -economic and cultural- these analyses challenge the idea that environmentalism among the upper-middle class primarily functions as a tool of distinction vis-à-vis lower classes. Indeed, the most strongly pro-environmental positions are associated with a symbolic rejection of the materialistic hedonism linked to economic capital. In other words, the pro-environmental orientation of the educated but not affluent upper-middle class emerges as a strategy of distinction more vis-à-vis the wealthy but less educated upper-middle class than in relation to lower classes (Flemmen, 2025). This interpretation is particularly useful not only for understanding consumption choices, but also for analyzing forms of entrepreneurial engagement that follow a similar logic: either through the translation of consumption choices into entrepreneurial action or through the direct projection of mechanisms of social distinction into entrepreneurial practices.

From this perspective, the theory of distinction can serve as a valuable complement to organizational studies for a deeper understanding of sustainable enterprises. While organizational studies have highlighted the importance of a specific hybrid configuration, whose model is strongly shaped by value orientations and entrepreneurial practices, the sociology of consumption helps to clarify the social foundations and generative mechanisms of such orientations.

In line with this theoretical framework, the first hypothesis that the article seeks to verify is that the key actor in new models of sustainable entrepreneurship is the upper-middle class, and within it the endowment of cultural capital plays a decisive role.

However, the role of the territorial context and the ways in which it may influence—either by fostering or hindering—the translation of a class-based eco-habitus into the spread of new forms of sustainable entrepreneurship remain underexplored. Even within organizational studies, reflection on the territorial embeddedness of hybrid enterprises remains relatively underdeveloped, despite the recognition of the role of certain contextual conditions such as relationships with stakeholders and the influence of formal and informal institutions (Muñoz and Cohen, 2017), as well as the availability of financial incentives (Talić et al., 2020), and notwithstanding some exceptions (Shrivastava and Kennelly, 2013; Cohen and Muñoz, 2015).

In particular, Shrivastava and Kennelly highlight the importance of the specific and potentially unique relationship that develops between firms and the contexts in which they operate, a relationship grounded in their *sense of place*. As the authors observe, “all organizational actions occur in places; understanding the motivations for sustainable or unsustainable organizational performance in those places makes all the difference” (Shrivastava and Kennelly, 2013, p. 97). By intersecting two key dimensions—orientation toward sustainability and organizational embeddedness in the territory—they define place-based enterprises as those firms that possess a strong sense of place and that, by pursuing a balance between financial-economic objectives and social and/or environmental goals, can be considered *place builders* rather than *place destroyers* (Shrivastava and Kennelly, 2013, p. 97). This represents a hybrid form of sustainable entrepreneurship that reconciles a market orientation with a value-oriented approach, among which is the promotion of the well-being of a specific territorial context.

From a different perspective -namely that of territorial inequalities and urban and regional transitions toward sustainability- the place-sensitivity of transition processes has been widely demonstrated. In particular, with reference to clean technologies, it has been shown that some territories face greater difficulties than others in initiating and sustaining new productive and economic models (Vale et al., 2024). On the one hand, it is commonly recognized that, in the face of global challenges, certain places—especially large cities and major urban areas- are at the forefront of transition processes, as they offer favorable conditions for innovation. On the other hand, it has been demonstrated that not all cities evolve at the same pace toward sustainable development models or in the same ways (Vale et al., 2024; Breglia and Sonzogno, 2026). Strongly dependent on contextual conditions, the capacity to mobilize in support of transitions tends to be more problematic in economically peripheral areas, due to pre-existing socio-economic and governance structures, the scarcity or fragility of local and cognitive resources, weaker connectivity, and the need for more substantial public intervention (Vale et al., 2024). In line with this perspective, in the Italian case it has been shown how the distribution of investment projects linked to the green transition within the framework of the National Recovery and Resilience Plan (PNRR) tends to reproduce pre-existing territorial capacities and development models rather than respond to environmental needs, not only in terms of intensity but also in terms of the quality of the trajectories and types of investment pursued (Breglia and Sonzogno, 2026).

Not only are weaker territories those with a lower capacity to pursue the transition; they are also more exposed to its costs. A Europe-wide reconstruction of the impact of policies adopted to tackle climate change shows a regional vulnerability to transition that reproduces existing inequalities (Rodríguez-Pose and Bartalucci, 2024). Indeed, weaker territories are those most exposed to the difficulties of the transition, not only because of their greater dependence on polluting energy sources -both as areas of production and consumption- but also due to their exposure to the risk of losing qualified human capital. In fact, there is a trend toward the regional specialization of green technologies and innovation that requires and attracts skilled labor, reinforcing existing flows of human capital mobility from weaker areas toward more developed ones (Rodríguez-Pose and Bartalucci, 2024).

The second hypothesis to be tested is therefore that, in peripheral contexts, the local environment plays a limited role in supporting innovative forms of sustainable entrepreneurship.

To pursue the research objectives and test the proposed hypotheses, this article draws on research conducted in the city of Palermo, a context that is particularly relevant for the purposes of this analysis. Palermo is a city with a stagnant economy in Southern Italy, one of the European macro-regions affected by the development trap (Rodríguez-Pose et al., 2024). Although it is a large city, it has not succeeded in developing the concentration of innovative and sustainable activities typical of larger metropolitan areas and more developed regions of the country. It is thus among the areas most vulnerable to the transition (Rodríguez-Pose and Bartalucci, 2024) and characterized by green transition trajectories that are less oriented toward capital-intensive and technologically complex projects (Breglia and Sonzogno, 2026).

In the past, Palermo represented the model of “development without autonomy” typical of Southern Italy, in which weak industrialization was partly compensated by an anomalous expansion of personal services, especially in public employment, supported by a political class seeking consensus and stability (Trigilia, 1992). This development model, unable to rebalance the labor market, has long been associated with persistent social vulnerability, high unemployment, and widespread informal work, while improvements in well-being have been concentrated among dependent middle classes, whose status was primarily constructed through forms of political mobilization aimed at access to public employment. Subsequently, the city experienced urban transformations linked to the redevelopment of the historic center (Azzolina, 2009) and to a late and more limited form of gentrification (Azzolina and Montemagno, 2021), partly overshadowed by the effects of touristification. In the post-Covid period, Palermo has followed the national trend of growth in low value-added sectors, such as construction, tourism, and personal services. However, it continues to suffer from the weakness of its labor market, which underlies migration flows of educated and skilled young people leaving the city in search of better employment opportunities.

In a context traditionally characterized by a low propensity for risk, underlying the historical weakness of the entrepreneurial class and, consequently, of the productive structure, the challenge is to understand what drives individual activation in the form of entrepreneurship, even before addressing the origins and motivations of innovative and transformative entrepreneurial action oriented toward environmental sustainability. What generates, in this context, the drive toward entrepreneurship? And what space can innovative and sustainable entrepreneurial forms realistically occupy?

Based on the definition of place-based enterprises (Shrivastava and Kennelly, 2013), this research develops a mapping and analysis of a sample of firms in the urban context of Palermo, where traits of a critical and educated middle class emerge supported by family resources, inspired by post-materialist and pro-environmental values, weakly integrated into the labor market yet unwilling to migrate. This group turns toward new forms of self-employment aimed both at income generation and at self-realization in creative, expressive, and lifestyle related terms associated with responsibility. These initiatives give rise to organizational forms in which environmental protection and attention to workers coexist with the need to generate income, albeit in tension with growth objectives.

The case study confirms what has already been highlighted in the literature on sustainable entrepreneurship regarding the importance of value orientation, as opposed to the instrumental rationality typical of the traditional entrepreneur, as well as the coexistence of multiple objectives. At the same time, the research makes three original contributions to the existing literature. The first consists of the development of a typology of sustainable entrepreneurship which, by articulating the concept of hybridity, makes it possible to distinguish different trajectories and to identify their social bases and orientations. Secondly, it demonstrates the usefulness of drawing on the theories of eco-habitus and social distinction—usually applied to consumers—to interpret the genesis of the most innovative forms of sustainable entrepreneurship in the case of Palermo. Thirdly, it highlights—with reference to commercial and low value-added sectors—the paradoxical drive toward new models of sustainability-oriented entrepreneurship that emerges from a stagnant labor market, in which opportunities for social and professional positioning for a critical and territorially rooted middle class are sought in innovative and responsible forms of self-employment.

## 3 Data and methods

The definition of the unit of analysis for the empirical investigation draws on existing conceptual categories and typologies in the literature. In particular, the study relies on the notion of place-based enterprises, identifying forms of entrepreneurship that simultaneously exhibit market orientation, environmental sustainability orientation, and territorial embeddedness (Shrivastava and Kennelly, 2013). Within the broader category of place-based enterprises, we then used sustainability entrepreneurship as an umbrella term (Halberstadt et al., 2024) to distinguish the different ways of integrating economic and sustainable objectives.

Given their overall relevance within urban economies—recently amplified by processes of gentrification and touristification—as well as their specific importance in the context of the case study, the analysis focused on personal-service enterprises primarily operating in the food, tourism, and circular economy sectors. These are small, low value-added firms, yet increasingly central to contemporary urban economic systems.

In the absence of an existing database on this type of enterprise, the identification of relevant cases required several preliminary steps. The first step consisted of an on-desk mapping of enterprises that aligned with the theoretical framework outlined above. Identification was carried out through online keyword searches indicative of environmentally friendly or eco-friendly economic activities (for example, food: organic products, zero km, zero-waste shops; tourism and mobility: cycle tourism, bike hotels, eco-friendly hotels; circular economy: reuse, recycling…) and the consultation of websites and social media platforms with national mapping of green economic activities (such as: Green City Map of Greenpeace, https://www.greenpeace.org/italy/storia/13764/e-online-la-nostra-eco-mappa-per-vivere-la-citta-in-modo-sostenibile/; Italia che Cambia, https://www.italiachecambia.org/mappa/, Rete Italiana Economia Solidale https://rete-ries.it/), with the aim of selecting organizations potentially meeting the research criteria. This phase was followed by field verification, which served both to confirm the actual presence of the identified activities and to assess their relevance to the study’s objectives. The integration of documentary analysis and direct observation enabled a more accurate delineation of the set of actors to be included in the interviews. The mapping was therefore oriented toward identifying: (1) economic activities characterized by different organizational forms, including firms and cooperatives but all engaged in the continuous production of goods and services for the market; (2) expressions of local entrepreneurship; and (3) initiatives featuring an environmental sustainability dimension, understood either as environmental protection—through, for example, the promotion of organic or low-impact production—or as territorial stewardship in an eco-sustainable perspective, through practices such as short supply chains, zero-kilometer sourcing, and the use of local resources.

For data collection, the study adopts a qualitative approach, employing semi-structured interviews as the most suitable method given the research questions and the analytical context in which the study is situated. This technique is particularly effective for exploring entrepreneurs’ value orientations, motivations, and perceptions. Likewise, the interpretive approach used in the analysis of the interviews is especially appropriate for examining new forms of sustainable entrepreneurship and their dynamics of development and growth within an urban context, a privileged field of observation in which networks and needs tend to foster sustainability-oriented innovation (Muñoz and Cohen, 2017).

In a second phase, participants were recruited from within the identified set of enterprises. Recruitment took place through direct contact or online communication and was subsequently supported by snowball sampling, whereby some interviewees acted as gatekeepers, facilitating the identification of additional participants. Fieldwork thus enabled a further refinement of the mapping. Out of a total of 30 actors contacted, 24 agreed to be interviewed.

The interview guide is structured into four thematic sections, designed to explore in an integrated manner the biographical, organizational, market-related, and institutional dimensions of the entrepreneurial experience. The first section focuses on reconstructing the interviewee’s biographical profile, with particular attention to family background and educational and professional trajectories. It also examines work values, the relationship between work and private life, perceived autonomy, and levels of job satisfaction. The aim was to capture, on the one hand, the social basis of value orientations and, on the other, how these shape the actor’s economic and organizational practices. The second section concerns the enterprise itself and investigates the genesis of the entrepreneurial idea, the role of the territory, the personal networks activated during the start-up phase, resource acquisition, the composition of the enterprise, and any evolution in its value orientation. The third section examines the firm’s position within its reference market, exploring competitive advantages, target clientele, the use of digital tools, supply networks, innovation practices, attention to sustainability, and prospects for growth, including expansion and scaling processes. Finally, the last section focuses on the institutional context, with particular attention to the role of local institutions and public policies in supporting or hindering the development of the entrepreneurial forms under investigation.

A total of 24 interviews were conducted, 22 of which took place in person and 2 online. At the request of some participants, dyadic interviews were also carried out. To ensure privacy, each interviewee was assigned a pseudonym, and the interviews are identified by a unique serial number, as reported in the table below (Table 1).

**Table 1:** List of interviewees.

Interview no.	Pseudonym	Sector	Date	Duration
1	Marta	Food	13.12.24	00:54:10
2	Luisa	Clothing	19.12.24	1:01:13
3	Carlo	Food	18.12.24	1:04:05
4	Luca_Gino	Food	22.01.25	01:21:31
5	Alfredo	Food	29.01.25	00:41:54
6	Anna	Circular economy	30.01.25	01:24:47
7	Fausto	Food	31.01.25	00:43:57
8	Lia_Cloe	Circular economy	06.02.25	01:06:37
9	Luigi	Food	18.02.25	01:42:14
10	Mara	Food	26.02.25	00:51:31
11	Agata	Food	04.03.25	01:01:49
12	Leo_Aldo	Sustainable tourism	11.03.25	01:17:12
13	Pietro	Tourism, sustainable mobility	16.03.25	00:49:58
14	Daniele_Olga	Food	17.03.25	01:12:00
15	Vera	Sustainable tourism	02.04.25	01:20:38
16	Manfredi	Food	02.04.25	01:02:00
17	Mario_Ciro	Food	08.04.25	00:54:57
17	Mario_Ciro	Food	28.04.25	00:58:29
18	Giulia_Marco	Circular economy, reuse	14.04.25	01:19:55
19	Isabella	Food	16.04.25	00:47:21
20	Davide	Sustainable tourism	17.04.25	00:57:11
21	Giada	Tourism	22.04.25	00:27:57
22	Aida	Food/circular economy	07.05.25	01:40:21
23	Alberto	Food	13.05.25	00:46:03
24	Viola	Circular economy	14.05.25	01:14:43

With the prior consent of the interviewees, all interviews were recorded and subsequently fully transcribed. The analysis of the texts followed a qualitative procedure structured in several stages.

In an initial phase (open reading), all transcripts were read in an exploratory manner in order to gain an overall understanding of the empirical material.

Subsequently (categorization), each interview was subjected to a second in-depth reading, aimed at assigning meaningful text units to conceptual categories. The formulation of the categories was based on the core themes of the interview guide (reflecting the research questions) and on analytical dimensions already highlighted in the literature.

In particular, attention was initially focused on the *field of the intention* dimension, as defined by Halberstadt et al. (2024), which was used as the primary analytical reference for a preliminary structuring of the collected information. Additional analytical categories were then added based on what emerged from the interviews. Specifically, the categorization proceeded through a distinction between thematic units related to entrepreneurial models, referring to the individual (e.g., career path, entrepreneurial choice, intentions, motivations), and thematic units related to organizational models, referring to the firm (e.g., firm size, financing mechanisms, supplier relationships, market positioning, sustainability practices).

The assignment of meaningful text units to both theoretically derived categories and categories suggested by the empirical material gave rise to an iterative process, during which the initial category of the *field of intentions* was adapted and modified, some initial categories were removed, and new categories were added and progressively refined.

Through successive refinements, the process made it possible to identify the existence of reciprocal links among certain categories, as well as the presence of three homogeneous sets based on recurring configurations, leading to the definition of a typology grounded in four analytical dimensions: *field of intention*, *prevailing motivation*, *organizational logic*, *role of the market*, and the three proposed patterns.

## 4 Sustainable entrepreneurship: the spatial distribution and types

### 4.1 The spatial distribution across the urban territory

**Figure 1:**
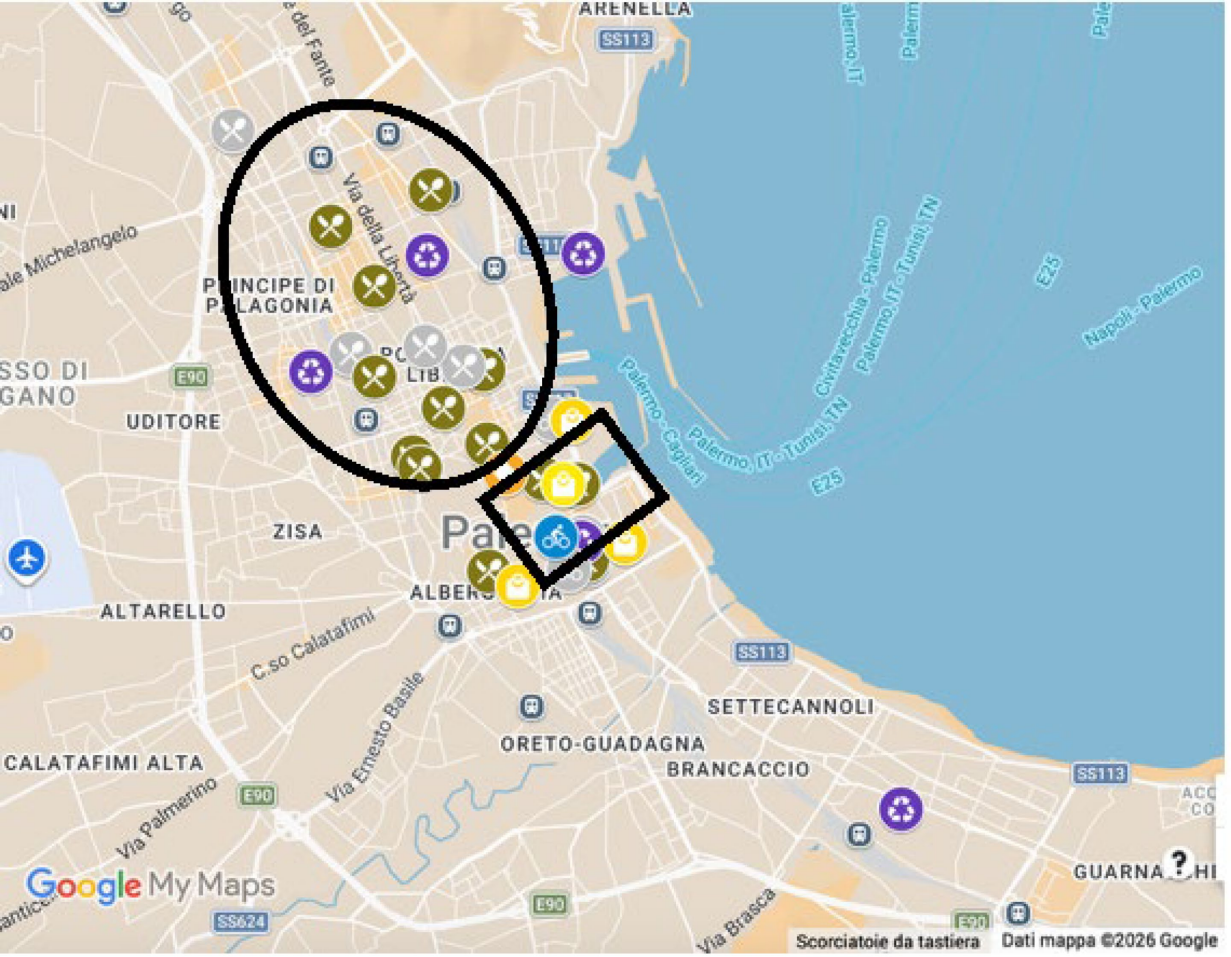
Custom geographical map of the study area, highlighting sustainable businesses, created by the authors using Google My Maps. *Map data: © 2026 Google*. This image is not covered by the Creative Commons license of this article and is used in accordance with Google’s Geo Guidelines.

A first finding concerns the spatial distribution of the sustainable enterprises mapped across the different urban areas (see Figure 1). The figure highlights their concentration in the historic city center (indicated on the map by the quadrangular shape) and along the northern axis (indicated by the oval shape), representing the expansion of residential neighborhoods traditionally inhabited by affluent social groups (Azzolina and Montemagno, 2021). This pattern suggests that sustainable entrepreneurial activities are, at least in part, connected to the refunctionalization and touristification of the historic center, while also reflecting their partial anchoring to the long-standing distribution of high levels of economic and cultural capital among the resident population.

### 4.2 Types of sustainable entrepreneurship

The analysis of the empirical materials collected reveals a varied and differentiated set of entrepreneurial experiences, demonstrating that speaking of sustainable entrepreneurship does not imply a homogeneous world of “do-gooders” (Halberstadt et al., 2024). Indeed, even within a sample that is, by design, entirely composed of sustainable and place-based firms, a spectrum of entrepreneurial models emerges, differentiated according to the entrepreneur’s history, motivations, and field of intention (Halberstadt et al., 2024), which, as we will see, also shapes organizational choices. A spectrum that varies along a continuum, where three different patterns can be (see Table 2) distinguished based on the intensity of certain traits relative to others, and where, although forms of hybridity may be present across all patterns, it is the strength of the emphasis on the prevailing traits that makes the difference.

**Table 2:** Types of sustainable entrepreneurship.

Key features	Pattern 1	Integrative	Pattern 2
Field of intention	Money driven	Hybrid	Social impact driven
Prevailing motivation	Profit	Profit and values oriented	Sustainable purpose
Organizational logic	Market-oriented	Adaptive	Values oriented
Role of the market	Main purpose	Mixed	Instrumental

At one end of the continuum (Pattern 1), we find the figure of the money-driven entrepreneur, primarily oriented toward achieving economic performance and investing in environmental sustainability as a strategic lever. Their predominant motivation is profit generation, pursued through the provision of sustainable products within an organizational structure that largely responds to market rules. Even this rational entrepreneur may seek to generate social value for the local area, yet organizational and entrepreneurial routines tend to remain focused on economic performance, and no particular attention is devoted to labor relations.

At the opposite end of the continuum (Pattern 2), we find the social entrepreneur, primarily oriented toward pursuing projects of social inclusion, within which environmental sustainability goals also find space, albeit in a secondary position. The prevailing motivation of social impact-driven entrepreneurs lies in the creation of social value, at times for employees and at other times for the external community. Although their aims are pursued through market activity, the enterprise adopts an organizational logic shaped more by the values that define it, above all, the integration of vulnerable individuals into the production routine.

Between these opposing poles we find entrepreneurial realities that we define as *integrative*, since in these cases the entrepreneurs’ economic rationality and value orientations merge and overlap; priorities become blurred, and organizational logics shift adaptively in an attempt to reach a difficult balance between market logic and the entrepreneurs’ value-driven orientations. Moreover, the integrative enterprise tends to be characterized by a higher frequency with which ecological concerns and attention to labor relations are combined within organizational and production routines.

#### 4.2.1 Pattern 1: Money-driven entrepreneur

To illustrate the first pattern, we examine two exemplary entrepreneurial trajectories. Both actors come from professional experiences within their family businesses, though in different sectors (respectively clothing and communication), and they enter sustainable food entrepreneurship equipped with managerial skills previously acquired elsewhere. Their move toward entrepreneurship is deeply rooted in their family histories. Their shift toward sustainable food, however, is the outcome of diversification strategies, though the underlying logic differs. In one case, the choice to enter the organic sector stemmed from an assessment of emerging market opportunities at a time when positioning within the organic segment could still be considered pioneering and the market appeared largely unexplored. As the entrepreneur recalls:

“I believed in it immediately, I believed in it right away and I immediately saw something worth investing in, and I must say we’ve been here for 35 years. We have doubled the national average for a business that usually lasts between 13 and 17 years!” (Fausto, int. 007, food).

In the other case, the entrepreneur’s motivations are more composite. Within an asset portfolio in which advertising service companies and real-estate firms represented the more traditional and speculative components of the business—“you buy, you rent, you sell… I do it, but it does not feel meaningful to me” (Manfredi, int. 016, food)—the entrepreneur initiates diversification processes into activities he himself describes as having a greater social impact, the most prominent of which concerns sustainable food. Here, the entrepreneurial logic diversifies and innovates, combining different motivational drivers. A first driver is the attempt to intercept emerging needs and new consumption practices:

“It was becoming clear that even in Palermo people appreciated not having to spend time shopping and cooking, but preferred to have lunch out, not go back home, go out…” (Manfredi, int. 016, food).

A second driver lies in supporting local producers through a commercial strategy based exclusively on offering zero-kilometer and regionally sourced products:

“We have been, and still are, a major showcase for Sicilian food because producers struggled to find places where their products could actually be consumed; many only export but incredibly, they are neither known nor consumed in Sicily” (Manfredi, int. 016, food).

An additional driver is the ambition to create a “food court” that could become a commercial hub capable of triggering the regeneration of a neighborhood located far from both the historic center and the main residential areas, and long characterized by the presence of illegality. As he explains:

“Today the neighborhood is known for the commercial hub; we have changed the collective imagination a bit… there are women who come here in the afternoon to knit, there are young people who come to study here as if it were a library… and we are happy, this is what the place is meant to be… we have achieved a social result, and I am pleased” (Manfredi, int. 016, food).

In Manfredi’s case, alongside economic rationality, we observe an entrepreneurial motivation that, although in the absence of a concrete search for added value for employees, extends to the pursuit of an outcome for the community. This entrepreneurial action does not appear to interfere with organizational routines, but rather reflects a quest for social recognition and for the legitimization of the entrepreneurial role. As he explains:

“The representation of the Italian entrepreneur in the 1990s and 2000s was distorted; the entrepreneur was seen as someone who steals or engages in wrongdoing, an image that in my view is very different from what entrepreneurs are in the majority of cases…” (Manfredi, int. 016, food).

However, when the commercial choice based on offering exclusively local and zero-kilometer products does not match consumer preferences, resulting in missed profits or even losses, the commercial offer adjusts to the demands of the market, incorporating more sought-after products and sacrificing the principle of environmental sustainability. As he explains:

“You lose money the first year, you lose money the second year, the third year, the fourth year… then comes the responsibility of the entrepreneur… I mean, you are not a charity… you have to return the investment to the shareholders; so that turnaround we made [abandoning zero-kilometer sourcing and local production and opening up to Coca-Cola] definitely improved our performance…” (Manfredi, int. 016, food).

It is worth noting that the diversity observed in the organizational and production routines of the two examples of food sustainable entrepreneurship is strongly influenced by the territorial location of the firm. The first is situated in a central, affluent residential area where, in the interviewee’s words:

“The people who live there… are people with a medium-high cultural background, and so we have always felt comfortable” (Fausto, int. 007, food).

The second is located in a peripheral residential neighborhood:

“We certainly paid the price for being in the outskirts; we addressed a social issue, and I am happy about that… today that goal has been achieved, but there is no doubt that I myself live… I am lucky enough to live in the center, I have studied, I know the difference between a local product… I have elements that help me make choices; not everyone has had this luck, this privilege, so it is clear that in the outskirts the cultural dimension is a bit different. Had we been in a central area, perhaps we would have improved our performance and who knows, maybe we would have remained faithful to that Sicilian integralism… Here we simply could not afford it…” (Manfredi, int. 016, food).

While the entrepreneurs’ declared passion sustains their motivation, solitude works in the opposite direction. This is, first of all, professional solitude, stemming from the lack of understanding and the absence of shared entrepreneurial energy among the original partners regarding the business idea. It is also institutional solitude, due to the lack of support from local institutions.

At the enterprise level, we observe that motivations and intentions more closely aligned with economic rationality, typical of the money-driven entrepreneur, tend to be associated with medium-sized firms characterized by a scaling orientation and financing mechanisms predominantly based on private capital. In this sense, financial autonomy emerges as a distinctive feature:

“I did it completely on my own, always taking the risk myself; I started the project, I financed it, I supported it…” (Manfredi, int. 016, food).

The relationship with the market appears primarily oriented toward growth and the maximization of sales:

“Food and fruit-and-vegetables… those are the things you have to start from…they are what can give you momentum…” (Fausto, int. 007, food).

Consistent with this orientation, supplier selection also ends up responding to criteria of efficiency and performance, even at the expense of eco-compatibility and the exclusive use of local products. As one interviewee following the same pattern notes:

“We prefer Italian products, yes, but for example we don’t turn down French products either…We are very interested in how Germany works with organic food, for instance, because it has a tradition in organics that is superior to ours…” (Alberto, int. 022, food).

#### 4.2.2 Pattern 2: Social impact-driven entrepreneur

In the second pattern, we encounter actors whose primary objective is the pursuit of a social project, with the market serving merely as the instrument for its realization. Strikingly, the word “enterprise” is largely absent from these interviewees’ vocabulary; economic activity is instead framed as a “project” of care directed toward social vulnerability, with environmental sustainability goals functioning as a complementary dimension. These entrepreneurs share with those in the previous model a comparable level of work experience, yet they display a distinctive common trait: a solid background in the social sector. All of them come from professional trajectories rooted in the world of social cooperation or from careers as social workers. Whereas the first group consisted of professional entrepreneurs, these are social professionals who use enterprise as a tool to pursue goals of a different nature, from the integration of migrants through circular-economy initiatives based on material recycling and maintaining a fully local supply chain (collection, sanitization, repair, and resale of textiles), to the labor reintegration of former inmates through zero-kilometer bakery enterprises, to social tourism organized around eco-compatible proposals without the use of vehicles, relying on recyclable materials and zero-kilometer catering. Here, self-entrepreneurship does not stem from a traditional propensity for business risk but rather from the desire to create pathways out of dependency and subordination for vulnerable groups—whether detained minors or migrants—based on the premise that employment is the foundation of social integration.

“Self-entrepreneurship, absolutely. Everyone must contribute something of their own and move beyond delegation, beyond the mindset of being dependent…” (Viola, int. 024, circular economy).

“We do not sell baked goods; we sell a project of social inclusion” (Agata, int. 011, food).

In other cases, the enterprise positions itself as a vehicle for the regeneration of marginal areas through the construction of a “shared beauty.” As one interviewee explains:

“During our walks we talked not only about the monuments but also about the social dimension of places, about the associations operating there, about what they were doing. We believed that those walks should help instill in people the desire for change, the desire for urban regeneration, the desire for a collective beauty, a collective good… to regenerate, to be with people, to understand the neighborhood’s problems in order to narrate them and fight for them: this is the principle of true sustainability…” (Davide, int. 020, sustainable tourism).

The social drive that sustains these entrepreneurial initiatives is closely linked to the social reading of places, an orientation that underpins a circular relationship between social and entrepreneurial experiences. These actors therefore stand in stark contrast to the solitude observed among entrepreneurs in the previous model: rather than operating in isolation, they are deeply embedded in community networks that they themselves help to construct. As one interviewee notes:

“Today we have an endless network of social organizations, so every one of our trips is planned within a certain social network, depending on how long the trip lasts…” (Vera, int. 015, sustainable tourism).

In this case, too, a recurring organizational configuration emerges among these entrepreneurial initiatives. Impact-driven enterprises are typically small to medium in size and rely on donations or public funding:

“We have made extensive use of foundations… of course there has also been an investment from our own foundation…” (Agata, int. 011, food).

This entrepreneurial model relies predominantly on local suppliers, reflecting its orientation toward the creation of shared value in which territorial impact plays a central role:

“Because we believe that in order to grow, it should not be only us growing, everything around us should grow as well…” (Davide, int. 020, sustainable tourism).

#### 4.2.3 Pattern 3: Integrative entrepreneur

Distinct both from the figure of the *money-driven* entrepreneur, whose priority is economic performance, and from that of the *social-impact-driven* entrepreneur, whose priority is social inclusion, the experience of the largest group of entrepreneurs in our sample seems to embody more fully than the others the traits of hybrid entrepreneurship. Despite the diversity of their professional histories, the actors examined here share a common trajectory: they turn to self-entrepreneurship after critical work experiences. At times, dissatisfaction becomes the primary motivation: a misalignment between personal orientations, lifestyle, and the standards imposed by their previous jobs, as in the case of Carlo, 44, an entrepreneur in the food sector.

“Yes, I graduated in economics in 2006… I did several jobs before starting this initiative… I did many things, really… since the jobs I had were not fulfilling… especially when I worked in insurance… and it wasn’t ethical for me to work in insurance (!)… I didn’t see it as something that belonged to me, I didn’t feel comfortable, it wasn’t my world… so I began thinking about doing something different, doing something of my own…” (Carlo, int. 003, food).

In other cases, dissatisfaction is tied to a sense of fatigue with work experiences pursued abroad, which fuels a desire to return home. As one interviewee recounts:

“At the end of the first Covid cycle, I decided to resign… not so much because of Covid -in fact, I had a permanent contract and everything-… But it was more due to my need to return to Italy; I genuinely felt the desire to come back to Palermo, because I was starting to miss everything… my homeland… my family… And also for work-related reasons, because I no longer felt satisfied; what I needed was for my creativity to be valued more…” (Luisa, int. 002, clothing).

At other times, we are confronted with the difficulties of entering the labor market through small, precarious, underpaid or, worse, entirely informal jobs:

“I was studying conifer pollen, but one day they made it clear that they would let me work but would not pay me… I was stupid, naïve, and young, and I thought that work should by definition be paid… I thought that work meant remuneration… but that is not the case… not here in Sicily, because that’s how it is… work is one thing and remuneration is another… they are two different worlds…” (Gino, int. 004, food).

This condition of instability and labor precarity can, in some cases, persist for long periods, as in the extreme case of Anna, 65, who describes the beginning of her current entrepreneurial activity together with her partners as follows:

“The four of us were flat on the ground, we had nothing left, but we were ready to start again…” (Anna, int. 006, accessories).

Individual mobilization in terms of self-entrepreneurship, rather than being directed toward caring for others, is here linked to self-realization, something that the lack of employment opportunities in a relatively stagnant urban context pushes toward entrepreneurial risk-taking. More broadly, it is the gap between the expectations of highly educated individuals and the concrete opportunities available to them that drives the turn toward self-entrepreneurship, which becomes the main, and at times the only, avenue for constructing personal well-being. While self-entrepreneurship emerges as an adaptive strategy in response to external conditions (lack of territorial opportunities), the choice to embrace sustainability is instead rooted in personal motivations, orientations, and lived experiences that shape the entrepreneurs’ way of being. In Carlo’s words, for instance, what clearly emerges is the transition from a critical consumer to a critical entrepreneur. His entrepreneurial idea stems from his own preferences, combined with a desire to generate environmental value:

“I have always been oriented toward organic products; they have always been part of my diet. Attention to nature, to natural and organic products, was beginning to emerge in Palermo, but there were not enough organic businesses in the city, and so I wanted, in my small way, to help make organic food better known.” (Carlo, int. 003, food).

Not unlike this, Gino’s entrepreneurial experience draws not only on his personal orientations but also on the competencies he has developed over time in relation to them. In his case, the distance between a commitment to high-quality products and the dominant market logic shaped by the standardized consumer becomes even more evident:

“You know, there are people who come in and ask for clementines [seedless citrus]. And he [referring to his business partner] still has the strength, the gentleness and the strength, to explain that we don’t sell clementines because they are a hybrid seed, something unnatural. I know people find it annoying to eat the seed… but the seed is life…” (Gino, int. 004, food).

For Pietro, everything originates from his passion for cycling which, as he puts it, “pushed me directly” toward both his entrepreneurial activity in the field of sustainable mobility and the creation of an urban blog focused on major mobility infrastructures, mass transit, and pedestrianization. Over time, this blog has become one of the main drivers of public debate in favor of pedestrianizing the historic center. His entrepreneurial trajectory thus emerges and develops in symbiosis with his civic mobilization, of which it is both an integral part and an expression. And although this is not a universal feature, some interviewees explicitly refer to a political orientation (not strictly party-political) that informs not only their biographical paths and their critical consumption practices, but also the way they do business, revealing a further shift from forms of political consumerism to forms of critical production:

“We were offered a tender to produce Puma’s Christmas bags… Imagine if we wanted to do it… You can imagine what we think about that… but of course we were very conflicted… luckily they rejected us, so in the end we didn’t do it. But you can clearly see that this is how our path works… every day… we are here discussing what to do…” (Gino, int. 004, food).

In Cloe’s experience, she recycles boat sails to produce sportswear, backpacks, and bags, the entrepreneurial choice stems not only from market considerations but also from a process of skill-building that follows, rather than precedes, the idea itself. In recounting the genesis of her business, she highlights an inductive process that moves from material to technique: the entrepreneurial idea emerges first, grounded in the creative intuition of repurposing discarded materials, and only later does the technical expertise take shape. As she explains:

“Our activity does not originate from a craft tradition, because there is no tradition, no savoir-faire, no know-how in tailoring or leatherwork or anything else; it starts from the materials. What this work was born from was a moment of profound change in which something more beautiful had to be reborn from a discarded material from the trash. The brand started like this: from fabrics, from markets, from remnants, from scrap PVC… it was about confronting the materials that arrived, and from that the product lines emerged… how to sew it, whether to sew it, whether to weld it… and then, little by little, the technique came. Let’s say I was largely self-taught until, at a certain point, I decided to invest in a prototyping course…” (Cloe, int. 008, circular economy).

But perhaps Olga’s entrepreneurial story is one where, more than any other, she places her personal evolution, her growth, her maturation as a consumer and in her relationship with food—“what I thought was right and what I wanted to offer”—at the center of her business idea. Olga states that she does not consider herself a restaurateur, but rather “a promoter of healthy food.” It’s the dramatic events of her private life that provide Olga with an understanding of the beneficial and healing properties of healthy food, hence the nature and characteristics of her entrepreneurial activity, which she describes as a venture for the promotion of well-being.

“Food is medicine, it’s healing, and it can help us live and feel better; hence all this research into food.” (Olga, int. 014, food)

That sustainable entrepreneurship is rooted in individual motivations does not mean, however, that these are introspective or self-referential experiences. On the contrary, there is a constant engagement with the external environment and with other entrepreneurial realities that sustains these trajectories and legitimizes their sense of “distinction”. As Olga notes when commenting on the growing attention to certain food products:

“What everyone today calls superfoods are, in the end, just… trends. Superfoods are a trend because they are simply fruits, vegetables, algae, seeds, legumes that are already valuable in themselves; they did not become superfoods, someone just gave them a label that works well for marketing…” (Olga, int. 014, food).

It is interesting to observe that this need for comparison and distinction appears rooted in a way of being even before it becomes a way of producing, as made explicit in the words of Marco, 50, who works in the field of circular economy and furniture restoration:

“If we go back to my youth, I always, let’s say, fought against my peers, whose greatest aspiration was to get a permanent job at the municipality, the regional administration … I fight every day against this mentality of taking as much as possible from the State, I want to be the protagonist of my own life. Of course I take risks, I take risks every day, because if I make a mistake, I pay for it, but it’s me, it’s me, I do what I want, I am free, I don’t have to answer to anyone, and that for me is priceless. And if I can do it through something I love, even better…” (Marco, int. 018).

The provision of quality through the expression of creativity, the pursuit of autonomy and freedom, and the affirmation of ethical principles guiding the entrepreneurial actions of these actors appears to be defined also in opposition to others. This need for distinction makes sensitivity to environmental issues part of their way of being even more than of their way of producing. It is worth noting that attention to the quality of working relationships, pursued in organizational practices, is likewise rooted in similar mechanisms of distinction. Previous experiences in their biographical trajectories and personal work histories have shaped a search for differentiation from a model of entrepreneurship—a way of being and producing—that is judged negatively and from which these entrepreneurs deliberately distance themselves. In this sense, the pursuit of distinction by these actors through their economic activity fosters the integration of sensitivity to environmental concerns with that to social issues and human relations, with particular attention to the creation of value for workers. This gives rise to several closely interconnected organizational consequences: a lack of interest in scaling up to larger business sizes, and a strong focus on the labor relationship. As Carlo explains:

“In restaurants it works like this… you spend your life in there… with this crazy split-shift system… which guarantees you nothing… You don’t have a life of your own… you simply can’t. Because you go in at nine in the morning and leave at midnight, with a break from three to five. What can you do? Nothing… it’s insane! So we proposed half-day shifts… either mornings or afternoons… so that people can have their own life and still work calmly. Basically, we have to guarantee serenity to anyone who works with us, because we have been on the other side… so we know very well that exhausting yourself all day long is not right” (Carlo, int. 003, food).

In line with these orientations, this type of hybrid enterprises, although growing, continue to maintain medium-small dimensions. Supplier selection is based on cost, quality, and ethical criteria. The market functions both as an end and as a means, accommodating a mix of competitiveness and collaborative practices. Their financing mechanisms combine private capital and public funds, with two elements standing out: on the one hand, the financial support and resources provided by the family of origin; on the other, the resources from *Io Resto al Sud* program. This is a public policy financed by the Italian state to support youth entrepreneurship in the southern regions by offering grants and subsidized loans for the creation or development of new economic activities. It is a funding instrument that appears repeatedly in the interviewees’ accounts and has played a far from marginal role in the emergence of these forms of sustainable entrepreneurship.

### 4.3 The dimension of environmental sustainability

Originally the central focus of the research and present by design across all enterprises in our sample, the dimension of environmental sustainability takes different forms within the three identified patterns. In the first pattern, it functions primarily as a tool for profit generation, in the second model it is secondary and accessory, sometimes instrumental, to social impact, to which all organizational resources are directed and to which even economic growth is implicitly subordinated, whether it is aimed at creating added value for employees or supporting community development.

Environmental sustainability is neither instrumental nor ancillary but is instead fully integrated into the entrepreneurial strategy of third-pattern enterprises, where the equilibrium point of economic growth emerges from a difficult compromise between environmental protection and the viability of the organizational model:

“We chose to give up 45% of our profit for a year… in order to offer a product of very high quality, extremely high, but at the same time hyper-accessible” (Marta, int. 001, food).

The full integration of environmental-sustainability objectives into production routines and organizational models supports the care of places, resources, and ecological rhythms, as well as short supply chains and the voluntary limitation of production volumes. These practices constitute a concrete response to the logics of overproduction and unlimited consumption:

“We harvested 40 lettuces? Then 40 it is; if you arrive and there are no more… there are no more!” (Gino, int. 004, food).

This commitment also includes the explicit effort to prevent forms of exploitation along the entire supply chain, as well as a strong attention to the quality of employment relations and to maintaining direct contact with producers:

“Making sure, for example, that there was no labor exploitation, that everyone was formally employed… that there was no precarious work… in the companies we buy from…” (Marta, int. 001, food).

“The eggs, for instance, we buy them from Mr. Mario, whom we know… we even know his hens, to whom he has given names…” (Carlo, int. 003, food).

Finally, the orientation toward sustainability also concerns production processes, where the recovery of discarded materials becomes a metaphor for a broader transformation in which what is considered “waste” is turned into value:

“What this work was born from was a moment of profound change in which something more beautiful had to be reborn from a discarded material from the trash…” (Cloe, int. 008, circular economy).

It is precisely thanks to the simultaneous integration of economic objectives, environmental protection, and social responsibility within their production routines and organizational model that these entrepreneurs embody the Triple Bottom Line (3BL) model more fully than others, while also experiencing its full complexity. Moreover, there is consensus that it is precisely this integrative logic that underpins sustainability and sustainable development (Martín-Rocañín et al., 2025).

## 5 Discussion and conclusions: a low-road to sustainability transition

Sustainable growth goals are at the center of the agenda of governments and institutions at all institutional levels; however, cities are at the forefront of this challenge. They can promote the transition through different trajectories, involving various public and private actors and across different sectors, ranging from capital-intensive industries to more widespread personal services. Emerging hybrid forms of entrepreneurship demonstrate that businesses themselves can play a role in promoting the transition through entrepreneurial projects that are more attentive to labor relations and environmental protection.

In line with the literature on sustainable entrepreneurship and with the various analytical categories already identified within it, this study has focused on sustainable and place-based enterprises located in a peripheral urban area of Southern Italy.

The focus on local entrepreneurship has made it possible to highlight the relevance of the sense of place in the economic activities examined, an aspect already noted in the literature and clearly confirmed by our analysis (Shrivastava and Kennelly, 2013). These firms are place-based in their ability to establish connections, build relationships, and participate in the tacit knowledge and shared meanings rooted in the local context, which they seek to preserve.

At the same time, the research has sought to fill some gaps in the literature on sustainable enterprises regarding the genesis of these new entrepreneurial models and the role played by context in conditions of peripherality, with the aim of understanding their generative mechanisms, distinctive features, and, ultimately, their potential in supporting green transition processes. The empirical materials collected and analyzed provide a number of insights that suggest original contributions to the ongoing debate.

The literature has long recognized the wide variability of sustainable and hybrid business models, many of which have already been classified within established frameworks and typologies (Halberstadt et al., 2024). However, the typology proposed in this work allows for two additional outcomes. First, the joint observation of entrepreneurial models and the organizational arrangements of firms made it possible to empirically demonstrate the assumption that, in start-ups and small enterprises, entrepreneurs shape organizational logics based on their own objectives and preferences (Schaltegger and Wagner, 2011). This is strongly supported by the evidence of identifiable patterns linking entrepreneurial models to the operational characteristics of firms. This reinforces the importance of deepening the analysis alongside organizational configurations also of entrepreneurial agency, through a better understanding of actors’ characteristics, including their social base, background, and prior experiences and skills.

Although all firms in the sample share sustainability profiles and may exhibit forms of hybridity, the first model is closer to the traditional model, where the logic of profit maximization prevails; the second approximates social enterprise; while it is in the third model that different value logics are most fully integrated (Halberstadt et al., 2024; Shrivastava and Kennelly, 2013). A second contribution of the proposed typology thus lies in focusing specifically on the characteristics of this integration within the third model.

While firms in the first model primarily emerge to respond to what are interpreted as market needs, and those in the second from an interpretation of unmet social needs, the entrepreneurial project of firms in the third model is instead linked to the provision of solutions hierarchized according to the entrepreneur’s values, preferences, and beliefs, subsequently seeking needs to address (Halberstadt et al., 2024). For these entrepreneurs, starting a business means building demand aligned with their preferences and values, “educating” consumers toward sustainable products, and “contributing to sustainability” (Halberstadt et al., 2024, p. 8).

Focusing on this aspect draws attention to still underexplored dimensions related to the entrepreneurial agency of sustainable forms of entrepreneurship.

The entrepreneurs in question generally possess high levels of education, substantial cultural capital, and a solid endowment of material resources, largely derived from their families of origin (economic assets, but also spaces, properties, and tools). They have developed value orientations and a pro-environmental ethos that guide their choices toward sustainability, typical of an integrated and relatively affluent middle class. In the narratives of these entrepreneurs, references emerge to consumption preferences, political values, and personal beliefs that indicate a transition from the active consumer (Arcidiacono, 2013)—sometimes political (Micheletti, 2010) or pro-environmentalist (Flemmen, 2025)—to the sustainability-oriented entrepreneur, who seeks social distinction from more predatory forms of entrepreneurship that are less attentive to environmental issues and social relationships. Entrepreneurial choices thus become part of a coherent life project, responding both to the need for economic integration and to the search for social legitimacy.

In this sense, the analysis of empirical materials allows us to confirm the hypothesis that the critical upper-middle class with high cultural capital is the driving force behind these forms of entrepreneurship.

However, these individuals are former workers and/or professionals with prior work experiences that were only partially satisfying or not satisfying at all, or with experiences of working abroad or in Northern regions. Strongly rooted in their local context, they find themselves constrained between an unwillingness to be geographically mobile and a lack of job opportunities: a bottleneck in which they experience a discrepancy between their expectations for life and self-realization and the working conditions available in a low-dynamism context. Escaping this bottleneck drives them toward the entrepreneurial choice, as a solution capable of generating personal well-being, which for individuals with these profiles is linked not only to income generation but also to self-expression, creativity, freedom, and responsibility.

With reference to the low-value-added sectors analyzed, peripheral conditions paradoxically act as a stimulus for the emergence of new entrepreneurial forms. Therefore, the second hypothesis is only partially supported, as the analysis highlights the contradictory role of the local context, particularly in production sectors characterized by low barriers to entry. On the one hand, spontaneous factors driving sustainable entrepreneurship—derived from market weakness—are evident; on the other hand, a lack of institutional support for these forms of entrepreneurship also emerges.

The entrepreneurial trajectories collected in the analyzed case study thus reveal both social and territorial determinants in the development of new forms of sustainable entrepreneurship. What emerges is an agency grounded more in social dispositions than in individual ones, linked to social groups that have developed socially responsible orientations and environmental sensibilities, but whose economic status appears eroded compared to the opportunities available within their families of origin. The weakness of the local labor market, combined with an unwillingness to be geographically mobile, represents a driving factor toward entrepreneurial risk-taking.

Ultimately, the case study suggests that forms of sustainable entrepreneurship in low-barrier-to-entry sectors can be interpreted as socially grounded adaptation strategies of the middle class to their context, taking the shape of a new mode of market mobilization that combines a distancing from the pure logic of profit and competition with the pursuit of self-realization.

The theoretical implications of these findings lie, first of all, in the need to adopt a multi-level approach to the study of sustainable entrepreneurship that integrates entrepreneurial action, organizational configuration, and territorial conditioning. It is not sufficient to identify the organizational configuration of the hybrid enterprise and the inherent tension between growth and the promotion of sustainability. It is also necessary to consider the territorial context within which the structure of constraints and opportunities, shaping the emergence and operation of firms, is embedded. Within this framework, the role of entrepreneurial agency can be interpreted. This does not coincide with reducing the phenomenon to individual dispositions, since actors respond not only to economic-territorial constraints (such as labor market conditions) but also to demands for recognition and social positioning (in relation to social groups), deploying the resources at their disposal (cultural capital and eco-habitus).

A further implication is that, rather than being viewed as a pure model endowed with its own intrinsic coherence, the hybrid enterprise should be studied as an adaptive solution, recognizing its variable, contingent, and evolving nature, including in terms of its transformative impact.

In the case study, we observed entrepreneurial experiences in low value-added sectors with a low level of organizational complexity, experiences that, in terms of their origins, characteristics, and productive sectors, appear in themselves incapable of driving systemic transition processes, let alone determining an economic repositioning of the territory or fostering specialization in green activities. However, they reflect a “social space” of market mobilization consistent with the promotion of sustainability in everyday life and in a widespread lifestyle. One could speak of a “low-road to transition,” emerging from spontaneous adaptations, but which highlights the need for greater institutional support.

Recognizing the specificities of these models of entrepreneurship implies, at the practical and policy level, the need to design targeted support measures and policies. As we have seen in the genesis of these experiences, a role has been played by financial instruments aimed at less dynamic areas, such as those provided by Io resto al Sud-Invitalia program. However, the hybrid nature of these enterprises calls for the development of incentives specifically tailored to their needs and calibrated to the requirements of the territories in which they operate.

Although the analysis focuses on a single case study, that of Palermo, the findings may also be relevant for understanding similar dynamics in other peripheral urban contexts. In particular, the interpretation of sustainable hybrid entrepreneurship as a socially embedded middle-class adaptation strategy may be extended to contexts characterized by limited economic opportunities, a weak industrial base, and stagnant labor markets. In such contexts, entrepreneurial initiatives may emerge not so much as drivers of systemic transformation, but rather as localized responses that combine economic survival, social recognition, and lifestyle aspirations.

Palermo can therefore be considered a relevant case study for exploring, both analytically and practically, a broader model of a “low-road to transition” in peripheral urban contexts. However, since the specific configurations of constraints, resources, and institutional support may vary significantly across contexts, comparative research in other peripheral cities will be necessary to verify the generalizability of these models and to further refine the theoretical framework.

## Data Availability

The raw data supporting the conclusions of this article will be made available by the authors, without undue reservation.
